# Tumor Growth Rate Predicts Pathological Outcomes in Breast Fibroepithelial Tumors: A Pilot Study and Review of Literature

**DOI:** 10.3390/cancers18020269

**Published:** 2026-01-15

**Authors:** Hisham F. Bahmad, Adriana Falcon, Abdallah Araji, Karem Gharzeddine, Youley Tjendra, Elena F. Brachtel, Natalie Pula, Nicole Brofman, Merce Jorda, Carmen Gomez-Fernández

**Affiliations:** 1 Department of Pathology and Laboratory Medicine, University of Miami Miller School of Medicine, Miami, FL 33136, USAcgomez3@med.miami.edu (C.G.-F.); 2 Department of Radiology, University of Miami Miller School of Medicine, Miami, FL 33136, USA; 3 Department of Radiology, Memorial Sloan Kettering Cancer Center, 1275 York Avenue, New York, NY 10065, USA

**Keywords:** fibroepithelial tumors, tumor growth rate, breast pathology, phyllodes tumor, fibroadenoma, diagnostic imaging

## Abstract

Background/Objectives: Fibroepithelial tumors (FETs) of the breast, including fibroadenomas (FAs) and phyllodes tumors (PTs), are among the most common breast masses encountered by breast radiologists and pathologists. Differentiating FAs from benign or borderline PTs can be challenging, especially on core biopsy specimens where sampling limitations obscure key histologic features. Although imaging techniques provide useful diagnostic context, their predictive accuracy for pathologic classification remains limited. Methods: We conducted a single-institution pilot study to assess whether tumor growth rate (TGR) derived from serial imaging could serve as a noninvasive correlate of histopathologic outcomes in FETs. Thirty-two patients with serial imaging and subsequent surgical excision (January 2020–May 2025) were analyzed. TGR, expressed as percentage volume increase per month, was calculated from diameter-based volumetrics. Results: The cohort included conventional FA (*n* = 10), cellular FA (*n* = 4), benign PT (*n* = 8), borderline PT (*n* = 6), and malignant PT (*n* = 4). Malignant PTs demonstrated significantly higher median TGRs (180.4%/month) and shorter imaging intervals (1.1 months) compared with other groups (*p* = 0.0357 and *p* = 0.005, respectively). These large effect-size differences suggest clinically meaningful growth dynamics. Conclusions: As a pilot, this study establishes foundational variance and effect-size estimates for powering a multicenter trial. If validated, TGR may provide an objective, noninvasive metric to enhance preoperative risk stratification and guide management of breast FETs.

## Simple Summary

Fibroepithelial tumors (FETs) of the breast, including fibroadenomas and phyllodes tumors, often present diagnostic challenges, particularly on core biopsy. Distinguishing benign from malignant subtypes is critical for guiding surgical management, yet conventional imaging provides limited predictive value. Our study is the first to systematically evaluate tumor growth rate (TGR) as a radiologic biomarker in FETs. We demonstrate that malignant phyllodes tumors (PTs) exhibit significantly higher TGR compared to fibroadenomas and benign/borderline PTs. These findings suggest that TGR, derived from routine serial imaging, offers a reproducible and noninvasive tool for early risk stratification. Integrating TGR into clinical practice could enhance preoperative decision-making, reduce diagnostic uncertainty, and improve patient outcomes by guiding timely surgical intervention.

## 1. Introduction

Fibroepithelial tumors (FETs) of the breast represent a heterogeneous group of biphasic neoplasms that can pose diagnostic challenges in clinical practice. This spectrum encompasses fibroadenomas (FA), benign phyllodes tumors (PTs), borderline PTs, and malignant PTs, each with distinct clinical management. While FAs and benign PTs are associated with similar and minimal recurrence risk, borderline and malignant PTs demonstrate higher potential for local recurrence and distant metastasis [1,2]. Accurate differentiation between FA and PTs (benign, borderline, or malignant) is clinically important, though distinguishing FAs from benign PTs may be less crucial given their similar low recurrence rates, overlapping recurrence profiles, and comparable clinical management [1,3]. However, identifying borderline and malignant PTs remains critical, as these require more aggressive surgical approaches including wide local excision with clear margins or even mastectomy. In addition, given the rarity of borderline/malignant PTs and conservative management of many FAs, adequately powered prospective cohorts are hard to assemble at a single center.

Current diagnostic approaches face significant limitations in predicting FET behavior and outcomes. Histopathological assessment, particularly when based on limited core needle biopsy (CNB) material, is often insufficient to reliably predict clinical behavior or differentiate between FET subtypes [1]. The clinical challenge of distinguishing FAs from PTs, particularly borderline and malignant subtypes, is indeed significant, as CNB has limited sensitivity for differentiating these lesions [4]. Similarly, conventional imaging modalities including ultrasound, mammography, and magnetic resonance imaging (MRI), while providing valuable lesion characterization, remain suboptimal in predicting the histologic subtype or clinical outcomes [5]. The NCCN guidelines specifically note that clinical suspicion of PT includes rapid growth as a key feature, alongside large size (>3 cm) and palpable mass [6].

Tumor growth rate (TGR) and tumor doubling time (TDT) have emerged as promising dynamic radiologic parameters with potential to predict tumor biology and clinical outcomes across various malignancies [7,8,9,10,11,12,13,14,15]. TGR quantifies the percentage increase in tumor volume over time, typically expressed as percent per month, obtained from sequential imaging studies using volumetric or diameter-based measurements [10,11,12,13,14,15]. This method has been validated as an early radiologic marker of treatment response and disease aggressiveness in neuroendocrine tumors, renal cell carcinoma, non-small-cell lung cancer, and breast cancer [7,8,9,10,11,12,13,14,15,16,17,18]. In breast cancer specifically, Lee et al. demonstrated that triple-negative and HER2-positive invasive breast carcinomas exhibited significantly faster TGR and higher rates of clinical upstaging during surgical wait times [11]. It is important to note that TGR represents a dynamic phenotypic biomarker reflecting tumor biology rather than an etiological determinant. Therefore, its predictive value is inherently context- and tumor type-specific and must be interpreted within the biological framework of each disease entity.

While TGR has demonstrated prognostic value in breast cancer—with studies showing that higher growth rates correlate with worse prognostic factors including higher histologic grade, lymphovascular invasion, and reduced disease-free survival—this metric has not been systematically evaluated in the FET spectrum [8,19,20]. In invasive breast cancer, TDTs vary widely even within molecular subtypes, ranging from approximately 105 days for grade III tumors to 353 days for grade II tumors [20]. More recent MRI-based studies show that luminal A breast cancers have an average TDT of 1126 days compared to 254 days for non-luminal subtypes [9]. Imaging studies also demonstrate that while FAs and PTs share similar morphologic features on conventional imaging, PTs are more likely to be larger than 3 cm, and have irregular shape, microlobulated margins, and higher BI-RADS categories [4,21].

Despite growing recognition of TGR’s clinical utility across multiple tumor types, its application in FETs remains unexplored. This knowledge gap represents a significant clinical opportunity, as an objective growth-based predictive marker could provide valuable adjunctive information, particularly when initial CNB yields equivocal results or imaging findings are non-specific. This study aims to evaluate TGR as a noninvasive radiologic marker for predicting histopathologic outcomes in breast FETs, with the potential to improve treatment decision-making and surgical planning through improved preoperative risk stratification. We purposely designed this work as a pilot, hypothesis-generating analysis to (i) establish operational feasibility of serial imaging TGR in routine practice and (ii) estimate effect sizes and variability needed for powering a definitive multicenter study.

## 2. Materials and Methods

### 2.1. Study Design and Sample Selection

We conducted a single-institution, retrospective, pilot study including patients diagnosed with breast FETs who underwent core needle biopsy followed by surgical excision (excisional biopsy, lumpectomy, or mastectomy) between 1 January 2020, and 31 May 2025. Inclusion criteria were strictly defined as follows: (1) availability of biopsy-confirmed histological diagnosis reviewed by board-certified breast pathologists; (2) availability of at least two serial imaging studies (magnetic resonance imaging (MRI), mammography, or ultrasound); and (3) both imaging examinations had to be of the same modality (MRI-MRI, mammography–mammography, or ultrasound–ultrasound). Patients who had incomplete imaging data, who lacked either biopsy or excision, or whose imaging modalities differed between evaluations (e.g., MRI to ultrasound) were excluded from the study.

### 2.2. Pathological Evaluation

Excisional specimens were reviewed by breast pathologists and classified into conventional FA, cellular FA, and benign, borderline, or malignant PTs, according World Health Organization (WHO) guidelines for breast tumors [22]. Detailed histologic parameters reviewed and validated included stromal cellularity, atypia, mitotic rate, tumor borders, presence of necrosis, and heterologous malignant elements.

### 2.3. Imaging Data and Tumor Growth Rate Calculation

**Figure 1:**
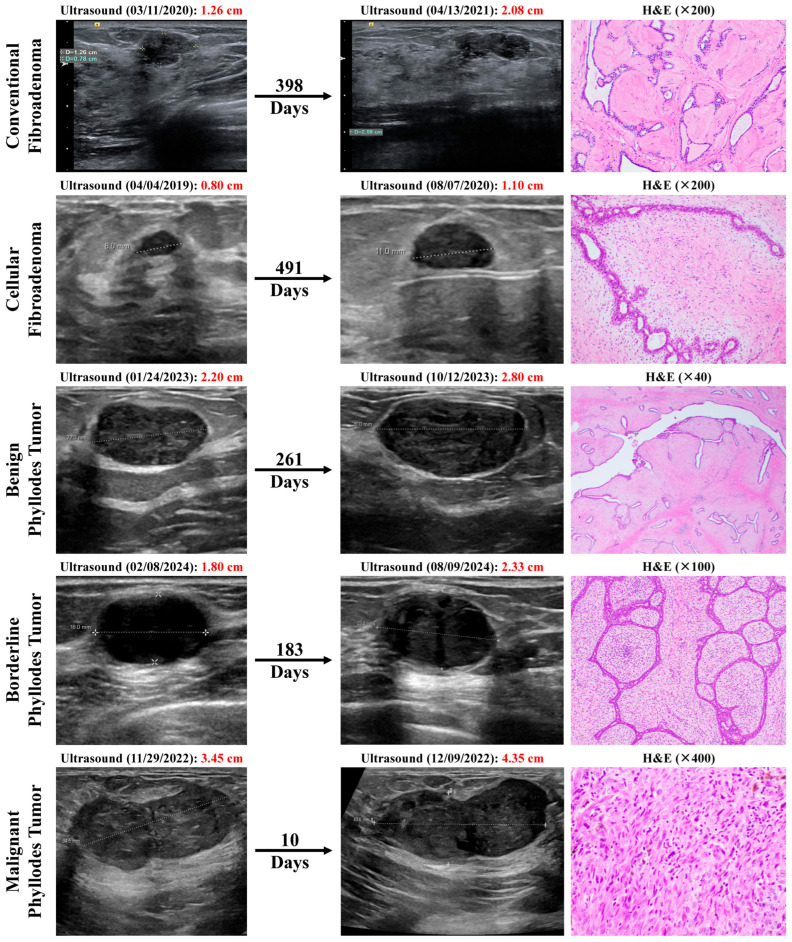
Breast ultrasound images showing interval increase in mass sizes over time in representative examples from our patient cohort of conventional fibroadenoma (FA), cellular FA, benign phyllodes tumor (PT), borderline PT, and malignant PT, and their corresponding hematoxylin and eosin (H&E) histopathological images on excision.

Imaging data, including tumor diameter, were collected and calculated from serial MRI, mammography, or ultrasound reports (Figure 1). All ultrasound examinations were performed as part of routine clinical care using standard high-frequency linear transducers approved for breast imaging. While transducer specifications (e.g., frequency range, manufacturer) were not explicitly standardized across all examinations, serial measurements for each individual patient were obtained using the same imaging modality, thereby limiting intra-patient technical variability. This pragmatic approach reflects real-world clinical practice and supports the feasibility of TGR assessment using routinely acquired imaging data.

**Figure 2:**
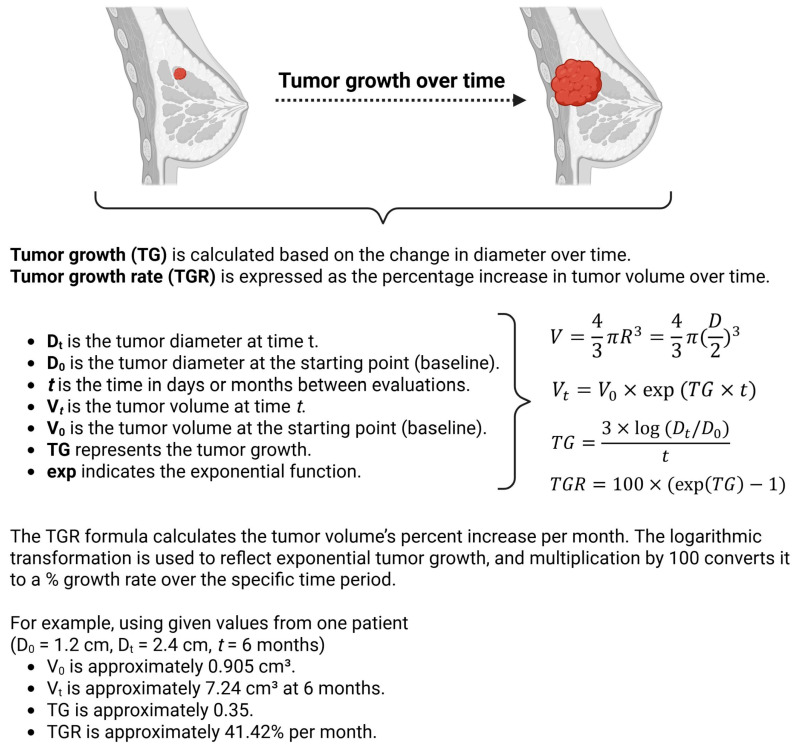
Schematic illustrating the calculations performed. Created in BioRender (2025).

Tumor size was defined as the longest diameter of the target lesion as previously described and as per the RECIST criteria [10,23] (Figure 2). The Response Evaluation Criteria in Solid Tumors (RECIST) is a standard way to measure how a cancer patient responds to treatment. A diameter-to-volume exponential transformation was used to approximate tumor kinetics while minimizing additional segmentation workload, making this approach critical for feasibility in a pilot study. Although PTs may exhibit irregular morphology, spherical approximation based on maximal diameter has been widely adopted in prior TGR studies as a pragmatic and reproducible approach when full volumetric segmentation is unavailable [10,12,13,14,15]. This trade-off prioritizes feasibility and inter-observer reproducibility in routine clinical settings.

Let *t* represent the time (in days or months) when the tumor is evaluated. Tumor growth rate (*TGR*) was calculated based on the exponential growth assumption. The tumor volume at time *t* (denoted as *V_t_*) can be expressed as

$$V_{t}=V_{0}\times exp(TG\times t)$$

where

• V*_t_* is the tumor volume at time *t*;
• V_0_ is the baseline tumor volume;
• TG is the tumor growth;
• exp indicates the exponential function.

To estimate the tumor volume (*V*) for the calculations, the tumor is approximated as a sphere, where the volume of a sphere is given by

$$V=\frac{4}{3}\pi R^{3}=\frac{4}{3}\pi {\left(\frac{D}{2}\right)}^{3}$$

Since the radius (*R*) is half of the tumor size (diameter *D*), we can rewrite *R* as *D*/2.

Consecutively, tumor growth (*TG*) is then calculated based on the change in diameter over time *t* as

$$TG=\frac{3\times log(D_{t}/D_{0})}{t}$$

where

• D*_t_* is the tumor diameter at time *t*;
• D*_0_* is the baseline tumor diameter;
• *t* is the time in days or months between evaluations.

To make the results clinically useful, *TGR* was expressed as the percentage increase in tumor volume over one month, using the transformation

TGR=100×(exp(TG)−1)

This formula calculates the tumor volume’s percent increase per month, where exp(*TG*) represents the exponential of the growth rate. The logarithmic transformation is used to reflect exponential tumor growth, and the multiplication by 100 converts it to a percentage growth rate over the specific time period.

### 2.4. Statistical Analysis

Data retrieved from medical charts were entered into a Microsoft Excel spreadsheet specifically designed for this study. Initial data management, cleaning, and descriptive analyses were performed in GraphPad Prism (version 10.5.0). Categorical variables (laterality, tumor site, biopsy diagnosis, margin status) were summarized as counts and percentages. Continuous variables (age at diagnosis, tumor gross size, initial tumor diameter [*D_0_*], follow-up tumor diameter [*D_t_*], time interval between imaging studies, and tumor growth rate [*TGR*]) were assessed for normality using the Kolmogorov–Smirnov and Shapiro–Wilk tests. Because all continuous variables were non-normally distributed, they were summarized as medians with interquartile ranges (IQR); means and standard deviations are also reported in Table 1 to complement medians/IQR and facilitate intergroup comparison.

Between-group comparisons for continuous variables across the five histologic categories (conventional fibroadenoma, cellular fibroadenoma, benign phyllodes tumor, borderline phyllodes tumor, and malignant phyllodes tumor) were performed using the Kruskal–Wallis H-test, followed by Dunn post hoc pairwise comparisons with Benjamini–Hochberg false discovery rate (FDR) control (Table S1). For categorical variables, several contingency-table cells had expected counts < 5 (including 0s), violating χ^2^ test assumptions; therefore, an exact, small-sample-valid permutation test of independence (Monte-Carlo approximation with 200,000 random label permutations; group sizes held fixed) was used to obtain *p*-values. This approach controls type-I error without reliance on large-sample approximations and is appropriate for sparse tables. All tests were two-sided with α = 0.05.

To further explore diagnostic performance in this pilot cohort, receiver operating characteristic (ROC) analyses were performed using TGR (%/month) to discriminate (i) borderline or malignant PTs versus other FETs and (ii) malignant PTs versus all other FETs (sensitivity analysis). Area under the ROC curve (AUC) was estimated using nonparametric ROC analysis in GraphPad Prism, while 95% confidence intervals for AUC and the optimal cut-point were estimated using nonparametric bootstrap resampling (Table S2). An optimal cut-point was identified by maximizing Youden’s J statistic (sensitivity + specificity − 1), and bootstrap 95% confidence intervals were calculated for the cut-point and associated sensitivity, specificity, positive predictive value (PPV), and negative predictive value (NPV) (Table S2).

## 3. Results

### 3.1. Patient Demographics and Tumor Characteristics

A total of 32 patients with breast FETs met the inclusion criteria and were included in the final analysis. The cohort was stratified by final histologic diagnosis into conventional FA (*n* = 10; 31.3%), cellular FA (*n* = 4; 12.5%), benign phyllodes tumor (PT; *n* = 8, 25.0%), borderline PT (*n* = 6, 18.8%), and malignant PT (*n* = 4, 12.5%). The clinicopathological characteristics of the patients are summarized in Table 1. The median age at diagnosis ranged from 35.0 years in conventional FA to 47.0 years in borderline PT, with no statistically significant difference among the groups (*p* = 0.2946). Tumor laterality and anatomical location showed no significant distributional pattern across the groups.

### 3.2. Core Needle Biopsy Concordance

The histologic diagnosis on core needle biopsy was mostly FET for most FAs and benign and borderline PTs but not malignant PTs, which included other diagnoses such as “PT, at least borderline” and “malignant neoplasm with sarcomatous differentiation, high grade.” Tumor gross size ranged from a median of 19.5 mm in cellular FA to 35.0 mm in malignant PT, but this difference was not statistically significant (*p* = 0.4718). Margin status on excision was reported only in PTs and did not significantly differ among the three PT groups (*p* = 0.685).

### 3.3. Radiological Growth Parameters

**Figure 3:**
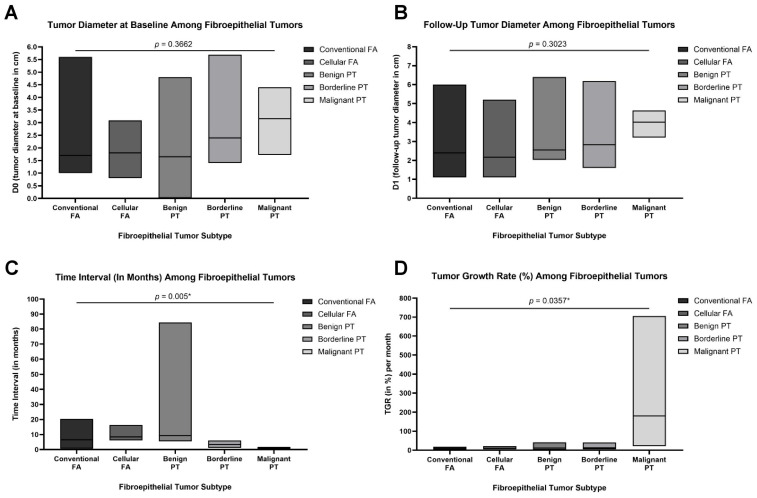
Box plots demonstrating key tumor metrics across the five fibroepithelial tumor subtypes: conventional fibroadenoma (FA), cellular FA, benign phyllodes tumor (PT), borderline PT, and malignant PT (total N = 32). (**A**) Baseline tumor diameter (D*_0_*) in centimeters measured radiologically at initial presentation. (**B**) Tumor diameter at follow-up (D*_t_*) in centimeters, measured at the time of surgical excision or latest imaging. (**C**) Time interval (in months) between initial and follow-up measurements, showing significantly shorter intervals for borderline and malignant PTs (*p* = 0.005). (**D**) Tumor growth rate (TGR, expressed as % per month), calculated using the change in tumor diameter over time, revealing significantly accelerated growth in malignant PTs compared to other subtypes (*p* = 0.0357). Data are presented as medians and floating bars (minimum to maximum). Statistical comparisons were conducted using Kruskal–Wallis tests followed by pairwise post hoc analyses where appropriate. Asterisks indicate statistical significance (* *p* < 0.05).

Radiologic assessments revealed increasing baseline (D*_0_*) and follow-up (D*_t_*) tumor diameters across the fibroepithelial spectrum, from FAs to malignant PTs. However, these differences were not statistically significant (D*_0_*: *p* = 0.3662; D*_t_*: *p* = 0.3023; Figure 3A,B). In contrast, both the time interval between serial imaging studies and tumor growth rate (TGR) demonstrated significant variation across the histologic groups. Specifically, the median time interval between imaging studies was shortest in malignant PTs (33.0 days; 1.1 months), compared to longer intervals observed in benign PTs (278.50 days; 9.28 months) and cellular FAs (252.50 days; 8.42 months). These differences were statistically significant (*p* = 0.005; Figure 3C). Moreover, malignant PTs had a markedly elevated median TGR of 180.42% per month—more than tenfold higher than any other group (*p* = 0.0357). The rising trend suggests that rapidly growing lesions are more likely to be malignant on final pathology (Figure 3D).

### 3.4. ROC and Optimal Cutoff Analyses

**Figure 4:**
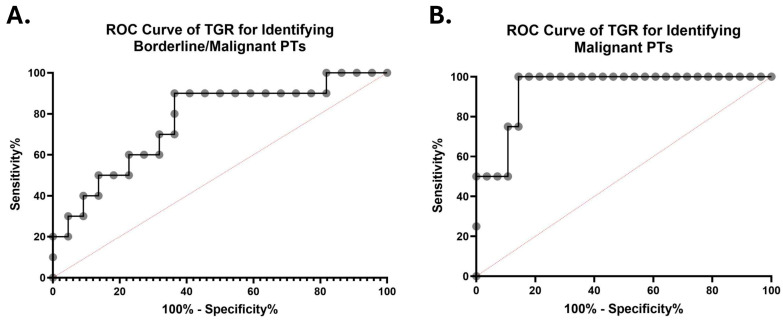
ROC curves evaluating the diagnostic performance of TGR (% per month) in discriminating borderline or malignant phyllodes tumors (PTs) from other fibroepithelial tumors (FETs) (**A**) and malignant PTs from other FETs (**B**). The AUCs were 0.764 (95% CI, 0.583–0.944) and 0.938 (95% CI, 0.846–1.000), respectively. The diagonal dashed lines represent no discriminative ability.

In a pilot ROC analysis, TGR demonstrated moderate discriminatory ability for identifying borderline or malignant PTs versus other FETs, with an area under the curve (AUC) of 0.764 (95% CI, 0.583–0.944; *p* = 0.0184) (Figure 4A). At the Youden-optimal cutoff (TGR > 11.67% per month), TGR achieved a sensitivity of 80.00% and a specificity of 63.64% for detecting borderline or malignant PTs. At this threshold, the positive predictive value (PPV) was 50.0%, while the negative predictive value (NPV) was 87.5%, indicating good rule-out performance (Figure 4A; Table S2).

In another exploratory analysis restricted to malignant PTs alone, TGR showed high apparent discrimination compared with all other FETs (AUC = 0.938; 95% CI, 0.846–1.000; *p* = 0.0052) (Figure 4B). At a Youden-optimal cutoff (TGR > 21.66% per month), TGR achieved a sensitivity of 75.00% and a specificity of 85.70% for detecting malignant PTs (Figure 4A; Table S2). However, given the small number of malignant cases (*n* = 4), these estimates were associated with wide confidence intervals and should be interpreted as hypothesis-generating rather than definitive.

## 4. Discussion

FETs of the breast, including fibroadenomas (FAs) and phyllodes tumors (PTs), are among the most frequently encountered breast masses in clinical practice [2,24]. While CNB often guides appropriate management, it may yield inconclusive results or suggest potentially concerning diagnosis, particularly with FETs [2]. In such cases, surgical excision is often required for definitive diagnosis. Often, when a breast lump is first evaluated radiographically, only a single measurement is available, providing no insight into growth kinetics; however, if radiologists document interval growth or rapid enlargement and communicate this to pathology, it adds critical context that may support the diagnostic and management process.

FA is the most frequent FET of the breast and the most common benign breast tumor. Phyllodes tumor is a biphasic tumor with a prominent intracanalicular component forming leaf-like structures lined by a double cell layer of epithelium and myoepithelium [1]. Preoperative differentiation between benign, borderline, and malignant PTs is essential, as the latter two subtypes have higher recurrence rates, 8.7% for borderline and 11.7% for malignant PTs, as shown in a study of 921 patients [25].

Current imaging modalities—ultrasound, mammography, and MRI—offer valuable insights, but their predictive capability for pathological outcomes remains limited [5]. Although PTs are more likely to be larger than 3 cm and have irregular shape, microlobulated margins, and higher BI-RADS categories compared to FAs, these static features have limited discriminatory power [4,21]. Recent advances in radiomics and deep learning may have shown promise; however, these approaches require specialized software and have not been widely validated in clinical practice.

Traditional imaging criteria such as size and density are limited in their ability to predict pathological outcomes. However, even simple comparative approaches—such as monitoring changes in tumor size over time using serial ultrasound—can provide both radiologists and pathologists with important clues about tumor behavior, especially in the absence of histologic certainty. Tumor growth dynamics offer a novel quantitative measure that may enhance the predictive accuracy of imaging studies.

TGR has emerged as a promising early radiologic marker that may provide insights into tumor behavior [12,15]. TGR, calculated from changes in tumor volume by imaging over time, has shown utility in predicting outcomes in various malignancies, including neuroendocrine tumors (NETs) and renal cell carcinoma [14]. The methodology for calculating TGR varies across studies, with important implications for interpretation and comparison. The most common approach uses the modified Schwartz equation to calculate tumor volume doubling time (TDT): TVDT = (*t* × log2)/log(V2/V1), where *t* is the time interval between measurements and V1 and V2 are initial and final volumes. Volume can be estimated from diameter measurements using the formula V = 4/3π(d/2)^3^ for spherical tumors, as in our study, though this introduces error for irregular lesions. More sophisticated approaches use three-dimensional volumetric segmentation on serial imaging, which provides more accurate measurements, particularly for irregular tumors.

A study on NETs revealed that TGR presents a strong, early radiological biomarker able to predict progression-free survival. Moreover, several studies have explored the relationship between TGR and treatment response, suggesting that it can be a valuable prognostic biomarker [8,9,10,11,12,13,14,15,16,17,18]. In breast cancer, a study evaluated the TGR of invasive carcinomas during wait times for surgery quantitatively using ultrasonography, showing that carcinomas with aggressive molecular subtypes (triple-negative and HER2-positive breast cancers) displayed faster TGR and more frequent upgrading of the clinical T stage [11]. Nevertheless, there are no studies evaluating TGR in FETs. In NETs, tumor proliferation is routinely assessed using Ki-67 and mitotic rates as part of the grading criteria; however, in breast FETs, while stromal mitotic activity is considered, immunohistochemical assessment of proliferation (such as Ki-67 index) is not routinely employed, highlighting an area for potential research and standardization.

To date, our study is the first to systematically evaluate TGR in FETs. In our cohort of 32 patients, malignant PTs exhibited significantly shorter imaging intervals (median 33 days, *p* = 0.005) and higher TGRs (median 180% per month, *p* = 0.0357) than benign or borderline subtypes. Other variables, such as age, baseline tumor size, and imaging measurements, did not significantly differ among groups. These findings suggest that static imaging alone is inadequate. Instead, tumor growth kinetics—quantified by TGR and the time interval between scans—demonstrate strong association with malignant histology (Figure 1). This supports the concept that rapidly growing FETs warrant heightened suspicion and may require prompt intervention.

We found a significant association between high TGR and malignant PTs, consistent with their known aggressive biological behavior. Our results align with similar findings in breast ductal carcinomas, where higher TGR correlated with unfavorable molecular subtypes and clinical upstaging [11]. Importantly, this is the first study to systematically apply TGR assessment to FETs, demonstrating its potential as a noninvasive marker of malignant potential where standard imaging lacks specificity. Standard imaging modalities such as ultrasound, mammography, and MRI are routinely used to assess breast masses; however, their ability to distinguish between benign and malignant FETs remains limited. Ultrasound is typically the first-line modality and is preferable for assessing lesion morphology and vascularity, while MRI offers more detailed tissue contrast and is particularly sensitive for detecting multifocality or lesion extent. Despite these advantages, both modalities fall short in reliably predicting biological behavior, especially in borderline and malignant PTs. By incorporating TGR into the preoperative evaluation—especially in cases where CNB results are indeterminate—clinicians may be better positioned to triage patients for timely surgical excision with consideration of surgical margins, thereby mitigating the risk of underdiagnosis and treatment delay in borderline or malignant PTs. Nevertheless, at this stage, TGR should be interpreted not as a diagnostic threshold but rather just as a decision-support metric. Markedly elevated TGR values may prompt consideration of expedited surgical excision or closer multidisciplinary review, particularly when core biopsy findings are indeterminate.

The ROC results in our study are exploratory and intended to provide preliminary diagnostic performance estimates (Figure 4). The bootstrap confidence intervals are wide, reflecting small subgroup sizes—particularly malignant PTs—and potential interval-dependent ascertainment. Nonetheless, the observed AUC and the high NPV at the pilot cut-point support the concept that markedly elevated TGR may function as a practical decision-support signal for expedited excision or multidisciplinary review, pending prospective validation.

Several studies have previously examined TGR in breast cancer using different methodologies (Table 2). Feldstein and Zelen et al. (1984) proposed a novel analytical approach to infer natural tumor progression in untreated breast carcinoma, identifying distinct tumor progression pathways and estimating average transition times between biological states [16]. Head et al. (1993) analyzed thermographic abnormalities in a large patient cohort, demonstrating that patients with faster-growing tumors were more likely to have abnormal breast thermograms and worse prognosis, underscoring a potential link between tumor biology and growth kinetics [17]. Kuroishi et al. (1990) calculated tumor doubling time (TDT) using data from mass screening programs and serial tumor size measurements, showing that the fastest-growing tumors such as solid-tubular carcinomas were associated with poorer prognosis, and TDT was significantly correlated with survival after adjusting for age, clinical stage, lymph node metastasis, and histologic type [7]. These studies, among others outlined in Table 2 [8,9,11,18], highlight the utility of TGR and TDT as potential early predictors of tumor behavior and long-term outcomes, and they support the application of similar principles in evaluating breast FETs.

A major limitation of our study is the small sample size (N = 32), which reflects the clinical practice and standard of care following which most benign FETs are managed conservatively. Indeed, this small, pragmatic cohort reflects real-world constraints (rarity of malignant PTs; conservative management of many FAs). The purpose was not definitive classification accuracy, but to demonstrate feasibility and quantify the expected separation of TGR distributions across histologies. Larger prospective studies are needed to validate TGR as a biomarker, ideally in conjunction with molecular and histological profiling (e.g., *MED12*, *TERT* promoter, *RARA* mutations) to enhance risk stratification. If replicated prospectively, a TGR-augmented workflow could flag rapidly enlarging FETs for expedited excision. In addition, although MRI may provide more precise volumetric assessment, ultrasound remains the most commonly used modality for longitudinal assessment of fibroepithelial tumors in clinical practice. The present study was not designed to compare measurement accuracy across imaging modalities; instead, modality consistency within individual patients was prioritized to ensure valid longitudinal growth assessment. In addition, it is noteworthy to mention that shorter imaging intervals in malignant phyllodes tumors likely reflect heightened clinical concern in response to rapid growth rather than intrinsic scheduling bias. Although TGR is mathematically normalized to time, interval-dependent surveillance remains a potential confounder and should be addressed prospectively in future studies.

## 5. Conclusions

This pilot study is the first to highlight TGR as a promising noninvasive, radiologic marker for assessing the biological behavior of breast FETs. Malignant PTs demonstrated significantly shorter median imaging intervals (1.10 months; *p* = 0.005) and higher TGR compared to other FET subtypes. While the shorter imaging interval likely reflects clinical response to rapid tumor growth—prompting earlier radiological reassessment—it should not be interpreted as an independent prognostic factor. Rather, it underscores the potential value of TGR in identifying aggressive lesions that may benefit from expedited evaluation and management.

Rapid growth is already recognized as a key clinical feature suggestive of PTs, particularly malignant subtypes, in current guidelines [6]. Traditional imaging methods that measure size alone have limited predictive value; incorporating TGR into the preoperative assessment could improve clinical decision-making by facilitating earlier and more extensive surgical intervention when warranted.

The biological plausibility of TGR as a discriminatory marker is supported by the extensive literature demonstrating that TGR correlates with aggressive features and worse outcomes in breast cancer. In invasive breast cancer, triple-negative and HER2-positive subtypes exhibit significantly faster growth rates compared to luminal A tumors, with corresponding differences in clinical upstaging during surgical wait times [9,11]. Similarly, in PTs, recent rapid tumor growth has been identified as a poor prognostic factor for malignant subtypes [26,27,28,29,30].

Although promising, these findings are limited by the small cohort size. Larger, prospective, multi-institutional studies are warranted to validate TGR and evaluate its integration with histopathological and molecular markers for enhanced risk stratification in FETs.

## Figures and Tables

**Table 1:** Table summarizing the clinicopathological characteristics of the 32 patients included in this study, stratified by final histologic diagnosis into conventional fibroadenoma (FA), cellular FA, benign phyllodes tumor (PT), borderline PT, and malignant PT.

	Total (N = 32) N (%)	Conventional FA (*n* = 10) *n* (%)	Cellular FA (*n* = 4) *n* (%)	Benign PT (*n* = 8) *n* (%)	Borderline PT (*n* = 6) *n* (%)	Malignant PT (*n* = 4) *n* (%)	*p*-Value
Demographics							
Age (years)							
Median (IQR)	38.5 (30.75–47.0)	35.00 (25.75–43.00)	37.5 (36.25–38.25)	37.0 (22.0–47.0)	47.0 (45.5–50.75)	41.0 (35.75–47.5)	0.2946 ^†^
Mean (±SD)	38.94 ± 11.99	35.20 ± 11.75	37.00 ± 2.16	36.63 ± 15.17	47.33 ± 7.66	42.25 ± 14.45	-
Pathological features							
Laterality							
Left	15 (46.87%)	5 (50.00%)	4 (100.00%)	1 (12.50%)	3 (50.00%)	2 (50.00%)	0.0824 ^‡^
Right	17 (53.13%)	5 (50.00%)	0 (0.00%)	7 (87.50%)	3 (50.00%)	2 (50.00%)	
Tumor site (clock position)							
1 o’clock	2 (6.25%)	1 (10.00%)	0 (0.00%)	1 (12.50%)	0 (0.00%)	0 (0.00%)	0.1986 ^‡^
2 o’clock	7 (21.87%)	4 (40.00%)	1 (25.00%)	1 (12.50%)	0 (0.00%)	1 (25.00%)	
3 o’clock	2 (6.25%)	1 (10.00%)	0 (0.00%)	0 (0.00%)	1 (16.67%)	0 (0.00%)	
4 o’clock	2 (6.25%)	0 (0.00%)	1 (25.00%)	0 (0.00%)	0 (0.00%)	1 (25.00%)	
5 o’clock	1 (3.13%)	0 (0.00%)	0 (0.00%)	0 (0.00%)	0 (0.00%)	1 (25.00%)	
6 o’clock	2 (6.25%)	1 (10.00%)	0 (0.00%)	0 (0.00%)	1 (16.67%)	0 (0.00%)	
8 o’clock	3 (9.37%)	1 (10.00%)	0 (0.00%)	2 (25.00%)	0 (0.00%)	0 (0.00%)	
9 o’clock	3 (9.37%)	0 (0.00%)	0 (0.00%)	1 (12.50%)	2 (33.32%)	0 (0.00%)	
10 o’clock	5 (15.63%)	0 (0.00%)	1 (25.00%)	3 (37.50%)	0 (0.00%)	1 (25.00%)	
11 o’clock	1 (3.13%)	0 (0.00%)	1 (25.00%)	0 (0.00%)	0 (0.00%)	0 (0.00%)	
12 o’clock	3 (9.37%)	2 (20.00%)	0 (0.00%)	0 (0.00%)	1 (16.67%)	0 (0.00%)	
Axilla	1 (3.13%)	0 (0.00%)	0 (0.00%)	0 (0.00%)	1 (16.67%)	0 (0.00%)	
Diagnosis on biopsy							
FA	5 (15.63%)	1 (10.00%)	1 (25.00%)	2 (25.00%)	1 (16.67%)	0 (0.00%)	<0.001 ^‡^*
FET	23 (71.87%)	9 (90.00%)	3 (75.00%)	6 (75.00%)	5 (83.33%)	0 (0.00%)	
Other	4 (12.50%)	0 (0.00%)	0 (0.00%)	0 (0.00%)	0 (0.00%)	4 (100.00%)	
Tumor gross size (greatest dimension in mm)							
Median (IQR)	25.50 (18.75–38.50)	22.00 (15.75–26.00)	19.50 (16.00–33.25)	30.50 (24.00–39.25)	25.00 (17.50–47.50)	35.00 (31.00–36.25)	0.4718 ^†^
Mean (± SD)	30.72 ± 17.11	25.70 ± 14.84	29.75 ± 27.23	35.38 ± 17.86	32.50 ± 19.63	32.25 ± 9.14	-
Margin status							
Negative	9 (56.25%)	-	-	3 (50.00%)	3 (50.00%)	3 (75.00%)	0.685 ^‡^
Positive	7 (43.75%)	-	-	3 (50.00%)	3 (50.00%)	1 (25.00%)	
Radiological features							
D*_0_* (tumor diameter at baseline in cm)							
Median (IQR)	1.90 (1.48–2.92)	1.70 (1.33–2.43)	1.80 (1.48–2.20)	1.65 (1.55–2.25)	2.40 (1.85–4.55)	3.16 (2.58–3.69)	0.3662 ^†^
Mean (± SD)	2.46 **±** 1.51	2.43 ± 1.73	1.87 ± 0.94	1.96 ± 1.35	3.14 ± 1.83	3.11 ± 1.12	-
D*_t_* (tumor diameter at time t in cm)							
Median (IQR)	2.68 (2.03–3.86)	2.39 (1.70–2.80)	2.17 (1.70–3.13)	2.55 (2.23–2.90)	2.83 (2.41–4.80)	4.03 (3.58–4.42)	0.3023 ^†^
Mean (± SD)	3.11 ± 1.54	2.81 ± 1.69	2.66 ± 1.78	2.98 ± 1.44	3.53 ± 1.83	3.97 ± 0.64	-
Time interval (in days)							
Median (IQR)	186.50 (55.00–313.25)	198.00 (91.25–390.25)	252.50 (222.50–325.25)	278.50 (192.5–632.75)	102.00 (45.50–163.00)	33.00 (16.75–48.25)	0.005 ^†^*
Mean (± SD)	308.50 ± 484.41	250.00 ± 193.22	295.24 ± 135.08	678.88 ± 859.67	105.33 ± 68.08	32.00 ± 20.64	-
Time interval (in months)							
Median (IQR)	6.22 (1.83–10.44)	6.60 (3.04–13.01)	8.42 (7.42–10.84)	9.28 (6.42–21.09)	3.40 (1.52–5.43)	1.10 (0.56–1.61)	0.005 ^†^*
Mean (± SD)	10.28 ± 16.15	8.33 ± 6.44	9.84 ± 4.50	22.63 ± 28.66	3.51 ± 2.27	1.07 ± 0.69	-
TGR (%) per month							
Median (IQR)	11.67 (5.90–19.25)	7.72 (3.72–11.50)	7.35 (5.90–12.04)	11.36 (7.14–19.83)	12.63 (11.65–15.59)	180.42 (25.84–426.52)	0.0357 ^†^*
Mean (± SD)	44.87 ± 133.55	8.19 ± 5.84	10.58 ± 7.78	15.59 ± 13.39	16.54 ± 12.51	271.93 ± 323.68	-

Abbreviations: cm: centimeters; D*_0_*: tumor diameter at baseline; D*_t_*: tumor diameter at follow-up; FA: fibroadenoma; FET: fibroepithelial tumor; IQR: interquartile range; mm: millimeters; n: number; PT: phyllodes tumor; TGR: tumor growth rate; %: percentage. ^‡^ Statistical comparisons among groups were performed using exact permutation test of independence (Monte-Carlo approximation with 200,000 random label permutations; group sizes held fixed) for categorical variables. ^†^ Statistical comparisons among groups were performed using Kruskal–Wallis H-test for continuous variables. Post hoc pairwise comparisons for continuous variables were performed using Dunn’s test with Benjamini–Hochberg false discovery rate (FDR) adjustment. Effect sizes (Cliff’s delta) with 95% bootstrap confidence intervals are provided in Table S1. Asterisk (*) indicates statistical significance at *p* < 0.05.

**Table 2:** Summary of studies assessing TGR in the breast cancer literature.

Reference	Cohort Size	Intervention or TGR Calculation Method	Follow-Up	Results and Conclusions
Feldstein and Zelen, 1984 [16]	692 breast cancer patients	A novel analytical approach was employed to reconstruct the natural progression of the disease as if no therapeutic intervention had occurred, enabling the estimation of the average time intervals between sequential changes in the biological parameters that define the disease’s natural history	Average around 8 years	• Tumor progression pathways were identified based on three prognostic factors: (A) axillary lymph node status, (B) tumor nuclear grade, and (C) presence of sinus histiocytosis • Combinations of these letters identify 12 possible states, ranging from the most favorable, A+B+C+ to the least favorable, A−B−C−
Head et al., 1993 [17]	126 deceased breast cancer patients, 100 breast cancer survivors, 100 controls	Assessment of breast thermography to correlate between the growth rate of tumors and their metabolic heat	Not clearly stated	• 88% of deceased had abnormal thermograms • Patients with faster-growing tumors were more likely to have abnormal breast thermograms and worse prognosis
Kuroishi et al., 1990 [7]	122 breast cancer patients	TDT based on mass screening intervals and tumor diameter progression	Time interval between measurements ranged from 2 weeks to 91 months	• The geometric mean TDT among all cases was 174 days • Solid-tubular carcinoma showed the fastest growth (126 days) and papillotubular carcinoma the slowest (252 days) • Shorter TDTs were associated with poorer prognosis • TGR remained a significant predictor of survival after adjusting for clinical stage, lymph node status, age, histologic type, and year of treatment
Yoo et al., 2015 [8]	957 breast cancer patients	SGR (%/day) calculation using two-time-point tumor sizes by US	Median follow-up period was 70.0 months (range, 0–139 months and mean TDT 14.51 days)	• TGR was higher when initial tumor size was smaller • Lymphovascular invasion, axillary lymph node metastasis, and higher histologic grade were significantly associated with higher SGR • SGR was significantly associated with DFS • High SGR was significantly associated with worse DFS in a subgroup at initial presentation • Tumor size > 2 cm
Lee et al., 2016 [11]	323 breast cancer patients	SGR (%/day) calculation using two-time-point tumor sizes by US	Median 31 days (range, 8–78 days) from initial imaging to surgery	• Triple-negative cancer had significantly higher SGR (1.003%/day) than HER2-positive (0.859%/day) and luminal cancers (luminal B, 0.208%/day; luminal A, 0.175%/day) • TGR significantly associated with molecular subtype
Fischer, 2025 [9]	204 patients with 208 invasive breast carcinomas	Doubling time calculated from MRI (average interval 21 months)	Up to 5 years of retrospective MRI analysis	• Doubling time: luminal A: 3.1 yrs; luminal B: 1.7 yrs; non-luminal: 0.7 yrs • Early detection intervals of 2 to 3 years for non-HR women using MRI and of 1 year for HR women reasonable
He et al., 2025 [18]	DB-03 study: 524 patients DB-04 study: 557 patients	Rate of tumor growth (g-score) estimated from exponential growth/decay models using CT-based tumor measurements	Until database lock of DB-03 (May 2021) and DB-04 (January 2022)	• Lower g-score with trastuzumab deruxtecan (T-DXd) vs. ado-trastuzumab emtansine (T-DM1) and treatment of physician’s choice (TPC) • g-score inversely correlated with OS and PFS

Abbreviations: cm: centimeters; CT: computed tomography; DFS: disease-free survival; HR: high-risk; MRI: magnetic resonance imaging; SGR: specific growth rate; TDT: tumor doubling time; TGR: tumor growth rate; US: ultrasound; %: percentage.

## Data Availability

The original contributions presented in this study are included in the article/Supplementary Material. Further inquiries can be directed to the corresponding author.

## Supplementary Materials

The following supporting information can be downloaded at https://www.mdpi.com/article/10.3390/cancers18020269/s1. Table S1: Post hoc pairwise comparisons of clinicopathologic and imaging variables across fibroepithelial tumor subtypes with false discovery rate (FDR)-adjusted *p*-values and effect sizes. Table S2: ROC-derived diagnostic performance metrics for TGR, including AUC, optimal cutoffs, and associated sensitivity, specificity, PPV, and NPV with confidence intervals (CIs).

## References

[1] Tan B.Y., Acs G., Apple S.K., Badve S., Bleiweiss I.J., Brogi E., Calvo J.P., Dabbs D.J., Ellis I.O., Eusebi V. (2016). Phyllodes tumours of the breast: A consensus review. Histopathology.

[2] Wiratkapun C., Piyapan P., Lertsithichai P., Larbcharoensub N. (2014). Fibroadenoma versus phyllodes tumor: Distinguishing factors in patients diagnosed with fibroepithelial lesions after a core needle biopsy. Diagn. Interv. Radiol..

[3] Cowan M.L., Argani P., Cimino-Mathews A. (2016). Benign and low-grade fibroepithelial neoplasms of the breast have low recurrence rate after positive surgical margins. Mod. Pathol..

[4] Zhang M., Arjmandi F.K., Porembka J.H., Seiler S.J., Goudreau S.H., Merchant K., Hwang H., Hayes J.C. (2023). Imaging and Management of Fibroepithelial Lesions of the Breast: Radiologic-Pathologic Correlation. Radiographics.

[5] Iranmakani S., Mortezazadeh T., Sajadian F., Ghaziani M.F., Ghafari A., Khezerloo D., Musa A.E. (2020). A review of various modalities in breast imaging: Technical aspects and clinical outcomes. Egypt. J. Radiol. Nucl. Med..

[6] Gradishar W.J., Moran M.S., Abraham J., Abramson V., Aft R., Agnese D., Allison K.H., Anderson B., Bailey J., Burstein H.J. (2025). NCCN Guidelines^®^ Insights: Breast Cancer, Version 5.2025. J. Natl. Compr. Cancer Netw..

[7] Kuroishi T., Tominaga S., Morimoto T., Tashiro H., Itoh S., Watanabe H., Fukuda M., Ota J., Horino T., Ishida T. (1990). Tumor growth rate and prognosis of breast cancer mainly detected by mass screening. Jpn. J. Cancer Res..

[8] Yoo T.K., Min J.W., Kim M.K., Lee E., Kim J., Lee H.B., Kang Y.J., Kim Y.G., Moon H.G., Moon W.K. (2015). In Vivo Tumor Growth Rate Measured by US in Preoperative Period and Long Term Disease Outcome in Breast Cancer Patients. PLoS ONE.

[9] Fischer U. (2025). Tumor Growth Rate of Luminal and Nonluminal Invasive Breast Cancer Calculated on MRI Imaging. Clin. Breast Cancer.

[10] Ferté C., Fernandez M., Hollebecque A., Koscielny S., Levy A., Massard C., Balheda R., Bot B., Gomez-Roca C., Dromain C. (2014). Tumor growth rate is an early indicator of antitumor drug activity in phase I clinical trials. Clin. Cancer Res..

[11] Lee S.H., Kim Y.S., Han W., Ryu H.S., Chang J.M., Cho N., Moon W.K. (2016). Tumor growth rate of invasive breast cancers during wait times for surgery assessed by ultrasonography. Medicine.

[12] Lamarca A., Ronot M., Moalla S., Crona J., Opalinska M., Lopez Lopez C., Pezzutti D., Najran P., Carvhalo L., Bezerra R.O.F. (2019). Tumor Growth Rate as a Validated Early Radiological Biomarker Able to Reflect Treatment-Induced Changes in Neuroendocrine Tumors: The GREPONET-2 Study. Clin. Cancer Res..

[13] He L.N., Zhang X., Li H., Chen T., Chen C., Zhou Y., Lin Z., Du W., Fang W., Yang Y. (2020). Pre-Treatment Tumor Growth Rate Predicts Clinical Outcomes of Patients with Advanced Non-Small Cell Lung Cancer Undergoing Anti-PD-1/PD-L1 Therapy. Front. Oncol..

[14] Dromain C., Sundin A., Najran P., Vidal Trueba H., Dioguardi Burgio M., Crona J., Opalinska M., Carvalho L., Franca R., Borg P. (2021). Tumor Growth Rate to Predict the Outcome of Patients with Neuroendocrine Tumors: Performance and Sources of Variability. Neuroendocrinology.

[15] Dall’Olio F.G., Parisi C., Marcolin L., Brocchi S., Caramella C., Conci N., Carpani G., Gelsomino F., Ardizzoni S., Marchese P.V. (2022). Monitoring tumor growth rate to predict immune checkpoint inhibitors’ treatment outcome in advanced NSCLC. Ther. Adv. Med. Oncol..

[16] Feldstein M., Zelen M. (1984). Inferring the natural time history of breast cancer: Implications for tumor growth rate and early detection. Breast Cancer Res. Treat..

[17] Head J.F., Wang F., Elliott R.L. (1993). Breast thermography is a noninvasive prognostic procedure that predicts tumor growth rate in breast cancer patients. Ann. N. Y. Acad. Sci..

[18] He P., Gambhire D., Zhou H., Ma X., Emura Y., Laadem A., Leung D., Bates S., Fojo A.T., Rixe O. (2025). Correlation between tumor growth rate and survival in patients with metastatic breast cancer treated with trastuzumab deruxtecan. Oncologist.

[19] Bhattarai S., Klimov S., Aleskandarany M.A., Burrell H., Wormall A., Green A.R., Rida P., Ellis I.O., Osan R.M., Rakha E.A. (2019). Machine learning-based prediction of breast cancer growth rate in vivo. Br. J. Cancer.

[20] Förnvik D., Lång K., Andersson I., Dustler M., Borgquist S., Timberg P. (2016). Estimates of Breast Cancer Growth Rate from Mammograms and its Relation to Tumor Characteristics. Radiat. Prot. Dosim..

[21] Rosenberger L.H., White R.L., Tafra L., Boughey J.C., Johnson N.M., Pass H.A., Boolbol S., Landrum K.M., Gao Y., Yao K. (2025). American Society of Breast Surgeons and Society of Breast Imaging 2025 Guidelines for the Management of Benign Breast Fibroepithelial Lesions. JAMA Surg..

[22] Tan P.H., Tse G., Shin S.J., Val-Bernal J.F. (2019). Chapter 3: Fibroepithelial tumours and hamartomas of the breast. WHO Classification of Tumours Editorial Board. Breast Tumours.

[23] Therasse P., Arbuck S.G., Eisenhauer E.A., Wanders J., Kaplan R.S., Rubinstein L., Verweij J., Van Glabbeke M., van Oosterom A.T., Christian M.C. (2000). New guidelines to evaluate the response to treatment in solid tumors. European Organization for Research and Treatment of Cancer, National Cancer Institute of the United States, National Cancer Institute of Canada. J. Natl. Cancer Inst..

[24] Marcil G., Wong S., Trabulsi N., Allard-Coutu A., Parsyan A., Omeroglu A., Atinel G., Mesurolle B., Meterissian S. (2017). Fibroepithelial breast lesions diagnosed by core needle biopsy demonstrate a moderate rate of upstaging to phyllodes tumors. Am. J. Surg..

[25] Bartels S.A.L., van Olmen J.P., Scholten A.N., Bekers E.M., Drukker C.A., Vrancken Peeters M., van Duijnhoven F.H. (2024). Real-world data on malignant and borderline phyllodes tumors of the breast: A population-based study of all 921 cases in the Netherlands (1989–2020). Eur. J. Cancer.

[26] Liu J., Li F., Liu X., Lang R., Liang R., Lu H. (2023). Malignant phyllodes tumors of the breast: The malignancy grading and associations with prognosis. Breast Cancer Res Treat.

[27] Di Liso E., Bottosso M., Lo Mele M., Tsvetkova V., Dieci M.V., Miglietta F., Falci C., Faggioni G., Tasca G., Giorgi C.A. (2020). Prognostic factors in phyllodes tumours of the breast: Retrospective study on 166 consecutive cases. ESMO Open.

[28] Mihai R., Callagy G., Qassid O.L., Loughlin M.O., Al-Hilfi L., Abbas A., Campora M., Hodi Z., Ellis I., Lee A.H.S. (2021). Correlations of morphological features and surgical management with clinical outcome in a multicentre study of 241 phyllodes tumours of the breast. Histopathology.

[29] Li J., Tsang J.Y., Chen C., Chan S.K., Cheung S.Y., Wu C., Kwong A., Hu J., Hu H., Zhou D. (2019). Predicting Outcome in Mammary Phyllodes Tumors: Relevance of Clinicopathological Features. Ann. Surg. Oncol..

[30] Turashvili G., Ding Q., Liu Y., Peng L., Mrkonjic M., Mejbel H., Wang Y., Zhang H., Zhang G., Wang J. (2023). Comprehensive Clinical-Pathologic Assessment of Malignant Phyllodes Tumors: Proposing Refined Diagnostic Criteria. Am. J. Surg. Pathol..

